# Multimodal transcriptomics reveal myCAF-derived collagen signaling associated with impaired NK-cell cytotoxicity and immune exclusion in pancreatic cancer

**DOI:** 10.1186/s12967-026-08237-4

**Published:** 2026-05-22

**Authors:** Lei Kong, Shengxin Gu, Mingyang Wang, Lili Gao, Xiaoling Song, Jun Gu

**Affiliations:** 1 Laboratory of General Surgery and Department of General Surgery, Xinhua Hospital Affiliated with Shanghai Jiao Tong University School of Medicine, No. 1665 Kongjiang Road, Shanghai, 200092 China; 2 Shanghai Key Laboratory of Biliary Tract Disease Research, No. 1665 Kongjiang Road, Shanghai, 200092 China; 3 Department of Pathology, Xinhua Hospital Affiliated with Shanghai Jiao Tong University School of Medicine, No. 1665 Kongjiang Road, Shanghai, 200092 China

**Keywords:** PDAC, myCAF, TaNK, Collagen signaling, Immune exclusion

## Abstract

**Background:**

Despite years of research, pancreatic ductal adenocarcinoma (PDAC) remains one of the deadliest cancers. A highly fibrotic, immunosuppressive microenvironment poses a significant barrier to effective immunotherapy. As key innate immune effectors of antitumor immunity, the functional status of natural killer (NK) cells within this environment remains poorly understood. This study aims to explore the role of stromal–immune interactions, particularly myofibroblastic cancer-associated fibroblasts (myCAFs) and NK cells, in shaping immune dysfunction in PDAC.

**Methods:**

We integrated spatial, single-cell and bulk transcriptomic analyses to characterize NK-cell heterogeneity and its cellular and molecular regulation in PDAC. CellChat-based communication analysis, spatial co-localization, and multiplex immunofluorescence were utilized to identify regulatory interactions and validate spatial associations.

**Results:**

A tumor-associated NK (TaNK) subset with impaired cytotoxicity was identified. myCAFs strongly interacted with TaNK cells through collagen signaling. Within ligand–receptor pairs, COL1A2–CD44 and COL1A1–LAIR1 served as dominant mediators linking stromal remodeling to impaired NK cell function. Spatial and histological validation confirmed their co-localization, while tumors co-enriched with myCAFs and TaNKs exhibited immune exclusion and poor response to immunotherapy.

**Conclusions:**

Our study demonstrates that collagen signaling from myCAFs impairs NK-cell cytotoxicity and promotes immune exclusion in PDAC. These findings advance the understanding of stromal–immune mechanisms underlying immunotherapy resistance and highlight that targeting the myCAF–NK axis may help improve the response to immunotherapy in PDAC.

**Supplementary Information:**

The online version contains supplementary material available at 10.1186/s12967-026-08237-4.

## Introduction

Pancreatic ductal adenocarcinoma (PDAC) accounts for approximately 90% of pancreatic cancers and remains one of the deadliest cancer types globally [1, 2]. Despite the limitations of conventional therapeutic modalities, there has been a surge of interest in exploiting immunotherapy as a promising avenue for intervention [3]. Regrettably, the applicability of immune checkpoint inhibitor (ICI) treatment remains limited to a minority subset of PDAC patients [4, 5]. Its poor prognosis primarily stems from dense fibrotic tissue within the tumor microenvironment (TME) and weak immune activity. These factors impede the entry of immune cells into tumor tissues and diminish the efficacy of immune checkpoint therapy. [1, 2]. The dense extracellular matrix (ECM) and extensive stromal–immune interactions are widely recognized as major barriers to effective anti-tumor immunity in PDAC [3–6], yet the specific cellular and molecular mechanisms underlying their suppressive effects remain incompletely elucidated.

Natural killer (NK) cells are key cytotoxic effectors in the innate immune system. They eliminate malignant cells through perforin and granzyme-mediated killing and secrete cytokines to coordinate immune responses. Their ability to directly target tumor cells without prior antigen sensitization makes them a crucial determinant of antitumor immunity, demonstrating highly promising therapeutic potential in cancer immunotherapy [7–9]. Recent single-cell analysis has identified a dysfunctional subset of NK cells termed tumor-associated NK cells (TaNK), characterized by diminished cytotoxic potential in the TME [10]. Mechanistic studys further revealed that LAMP3^+^ dendritic cells act as key regulators mediating this NK cell functional suppression. However, PDAC exhibits unique fibrotic, stroma-dominant structural features, and the cellular drivers of NK cell dysfunction in this context remain unclear.

To address this issue, we employed an integrative multimodal transcriptomic approach combining spatial, single-cell and bulk transcriptomic analyses to systematically investigate the dysfunction of NK cells in the TME of PDAC. Using large-scale pancreatic cancer single-cell transcriptomics data, we identified a TaNK subset characterized by reduced cytotoxicity. Mapping intercellular communication network revealed strong interactions between fibroblast subtypes—particularly myofibroblastic cancer-associated fibroblasts (myCAFs)—and TaNK cells via collagen-related signaling. The COL1A2–CD44 and COL1A1–LAIR1 ligand–receptor pairs emerged as key mediators connnecting stromal remodeling to NK cell dysfunction. Spatial transcriptomics and multiplex immunofluorescence further validated the spatial colocalization and functional association between myCAFs and TaNK cells. Immuno-landscape analyses revealed that tumors co-enriching these two cell populations exhibited an immune-excluded phenotype and decreased immunotherapy responsiveness.

This study systematically elucidates a stromal–immune regulatory axis in PDAC, demonstrating for the first time that myCAF-derived collagen signaling drives NK cell dysfunction and immune exclusion, providing a mechanistic insight into the immunotherapy resistance of this malignant tumor. Understanding this stromal–immune interaction may offer potential therapeutic opportunities, as targeting the myCAF–NK cell axis could help restore antitumor immunity and ultimately improve the clinical management of PDAC.

## Methods & materials

### Data acquisition

The summary association results for NK cells-related immune traits were extracted from a large-scale GWAS study [11]. The GWAS summary statistics of pancreatic cancer were selected from FINNGEN (https://www.finngen.fi/en) [12]. The single-cell RNA sequencing data were obtained from eight independent public datasets. Among them, CRA001160 was retrieved according to reference [13], while the remaining seven datasets (GSE155698, GSE212966, GSE242230, GSE154778, GSE156405, GSE194247 and GSE235449) were accessed through the GEO database (http://www.ncbi.nlm.nih.gov/geo). Spatial transcriptomics data was also sourced from this repository (GSE235315, GSE272362). Bulk transcriptomics data for PDAC were obtained from TCGA (https://xena.ucsc.edu), GSE57495 and GSE71729 datasets. Additionally, dataset for predicting immunotherapy responses was explored using the IMvigor210 cohort (http://research-pub.gene.com/IMvigor210CoreBiologies).

### Mendelian randomization analysis

Mendelian randomization (MR) is a method utilizing genetic variation as instrumental variables to estimate causal relationships between target exposures and outcomes. It has been widely applied in studying causal associations among biological variables [14, 15]. This study employed a two-sample Mendelian randomization analysis to uncover the causal relationship between NK cell-associated immune traits and pancreatic cancer. NK cells-related immune traits served as the exposure, while the pancreatic cancer as the outcome variable, and single nucleotide polymorphisms (SNPs) as the instrumental variables. The two-sample MR was theoretically grounded in three core assumptions (i) the IVs exhibits a strong association with NK cells-related immune traits (ii) the IVs influence pancreatic cancer risk solely through their effect on NK cells-related immune characteristics (iii) the IVs are independent of other confounding factors. Five MR methods including inverse-variance weighted (IVW), MR Egger, simple mode, weighted median, and weighted mode were employed to investigate the causal relationship between NK cells-related immune features and pancreatic cancer based on IVs effects. IVs associated with pancreatic cancer risk were identified using a threshold of *p* < 1 × 10^−5^, consistent with previous studies [15, 16]. “mr_heterogeneity”, “mr_pleiotropy_test”, and “mr_leaveoneout” functions were used to analyze heterogeneity, pleiotropy, and sensitivity respectively. Two-sample MR analysis were performed with R package “TwoSampleMR”.

### scRNA-seq Data Analysis

The single-cell RNA sequencing data was converted into Seurat objects using the “Seurat package” in R software [17]. To ensure data quality, cells with mitochondrial gene content exceeding 25%, fewer than 10 detected genes, or fewer than 200 expressed genes were excluded from subsequent analyses. Batch effects were corrected using the Harmony method (v1.2.0), and Seurat objects were integrated into a unified dataset [18]. Principal component analysis (PCA) was then employed for dimensionality reduction, followed by clustering cells using the FindNeighbors and FindClusters functions. Data visualization was achieved through the Uniform Manifold Approximation and Projection (UMAP) algorithm. Cell type annotation was primarily based on results from the SingleR package and previously established marker genes.

### Spatial transcriptomics analysis

Spatial transcriptomics analysis was primarily performed using the “Seurat” R package, Spots with fewer than 200 unique features were filtered as low-quality, and the NK cell cytotoxicity score was calculated by the AddModuleScore function in Seurat with default parameters. Spatial spot deconvolution was performed using the cell2location package (version 0.1.4) [19]. This analysis integrated reference single-cell datasets encompassing five fibroblast subclusters and five NK-cell subclusters sourced from PDAC samples. The regression-based model was trained under default settings to achieve optimal accuracy in reconstructing spatial cell compositions. The final cell2location model was deployed with the parameters N_cells_per_location = 20 and max_epochs = 30000. Colocalization signal calculation followed those described in the previous research [20].

### Construction and validation of the prognostic signature

Through univariate Cox regression, we examined the transcriptomic and survival data of 137 PDAC patients from the TCGA database. Results revealed that NK cell specificity genes significantly associated with prognosis in this cohort (*p* < 0.05). Subsequently, the prognostic genes were subjected to the Least Absolute Shrinkage and Selection Operator (LASSO) Cox regression [21]. The selcected key genes were integrated into a multifactorial Cox regression analysis, and a risk model was conducted by linearly combining gene mRNA expression levels and their corresponding risk coefficients. Afterwards, patients were ultimately stratified into low-risk and high-risk groups based on the median cutoff value. Kaplan-Meier curves were used to evaluate overall survival (OS) differences between the two groups. Time-dependent ROC curves were generated using the “survivalROC” package in R software, and the area under the curve (AUC) was calculated to evaluate the prognostic efficacy of this NK cell marker gene signature in PDAC patients. Independent validation based on a GEO cohort further confirmed the predictive capability of the prognostic signature.

### Pathway and function enrichment analysis

The R packages “ClusterProfiler” and “org.Hs.eg.db” were used to perform the analyses of The Gene Ontology (GO), Kyoto Encyclopedia of Genes and Genomes (KEGG), and Gene Set Enrichment Analysis (GSEA).

### Cell-cell infiltration characterization and correlation analysis

CIBERSORTx was used to generate a reference signature matrix from the single-cell RNA-seq data, enabling estimation of cell-type proportions in bulk RNA-seq and microarray datasets [22]. Default parameters were employed throughout the analysis. Spearman’s correlation analysis assessed associations between cell-type infiltration proportions. Visualization was performed using the R package corrplot (v0.92), with a significance threshold set at *p* < 0.05.

### Survival analysis

The Survival (v3.5.5) and Survminer (v0.4.9) packages in R were used to perform survival analysis. Kaplan–Meier survival curves were generated via the survfit function, and the two-sided log-rank test was applied to compare survival curves.

### Cell-to-cell communication analysis

CellChat (v1.6.1) was used to infer cell-cell interactions and construct regulatory networks based on ligand-receptor interactions [23]. Interactions present in fewer than 10 cells were excluded. The netVisual function visualized interaction patterns. For pathways containing multiple ligand-receptor pairs, the netAnalysis_contribution function determined and displayed the contribution of each ligand-receptor pair to the pathway.

### Multiplex immunofluorescence staining

Pancreatic cancer tissue specimens were obtained from Xinhua Hospital, Shanghai Jiao Tong University School of Medicine. Primary antibodies used in this study included anti-CD56 (Proteintech, 14,255–1-AP, 1:4000), anti-CD3 (ABWAYS, CY5581, 1:1000), anti-GZMB (Abcam, ab255598, 1:3000), and anti-Periostin (Abcam, ab215199, 1:1000). Multiplex immunohistochemistry (mIHC) staining was performed using the Multiplex IHC Kit (RC0086-45, Recordbio) according to the manufacturer’s protocol. Tissue sections were first deparaffinized and rehydrated through graded xylene and alcohol solutions, followed by antigen retrieval and blocking procedures. Sections were sequentially incubated with primary and secondary antibodies and labeled with fluorescent dyes. Cell nuclei were stained with DAPI for 5 minutes and mounted with antifade mounting medium. Stained slides were scanned using a multi-channel fluorescence imaging system.

### Immunotherapy response prediction

Mutation data were sourced from the TCGA database. Tumor Mutational Burden (TMB) was quantified using the “maftools” R package. PDL1 mRNA expression data were derived from the TCGA pancreatic cancer cohort. To characterize T Cell Receptor (TCR) libraries, we used the Richness and Shannon diversity index. Richness quantified the unique TCR count per sample, whereas the Shannon Diversity Index elucidated the relative abundance across diverse TCRs. Enrichment and Shannon Diversity Index values for TCRs, along with Single Nucleotide Variant (SNV) neoantigen data, were obtained from a comprehensive pan-cancer mapping study [24]. The Tumor Immune Dysfunction and Exclusion (TIDE) score, an algorithm simulating key mechanisms of tumor immune evasion, such as T-cell dysfunction and T-cell rejection, provides a basis for predicting the responses of ICI in cancer patients(http://tide.dfci.harvard.edu). Furthermore, to validate the predictive utility of cell infiltration for immunotherapy responses, we collected transcriptomic and treatment response data from 348 bladder cancer patients in the IMvigor210 immunotherapy cohort.

### Statistical analysis

Data analysis and figure generation were performed using the R software (version 4.2.1, http://www.R-project.org). Statistical significance was defined as *p* < 0.05.

## Results

### Mendelian randomization and spatial transcriptomics identify NK-cell cytotoxic dysfunction as a risk-linked feature in pancreatic cancer

**Fig. 1:**
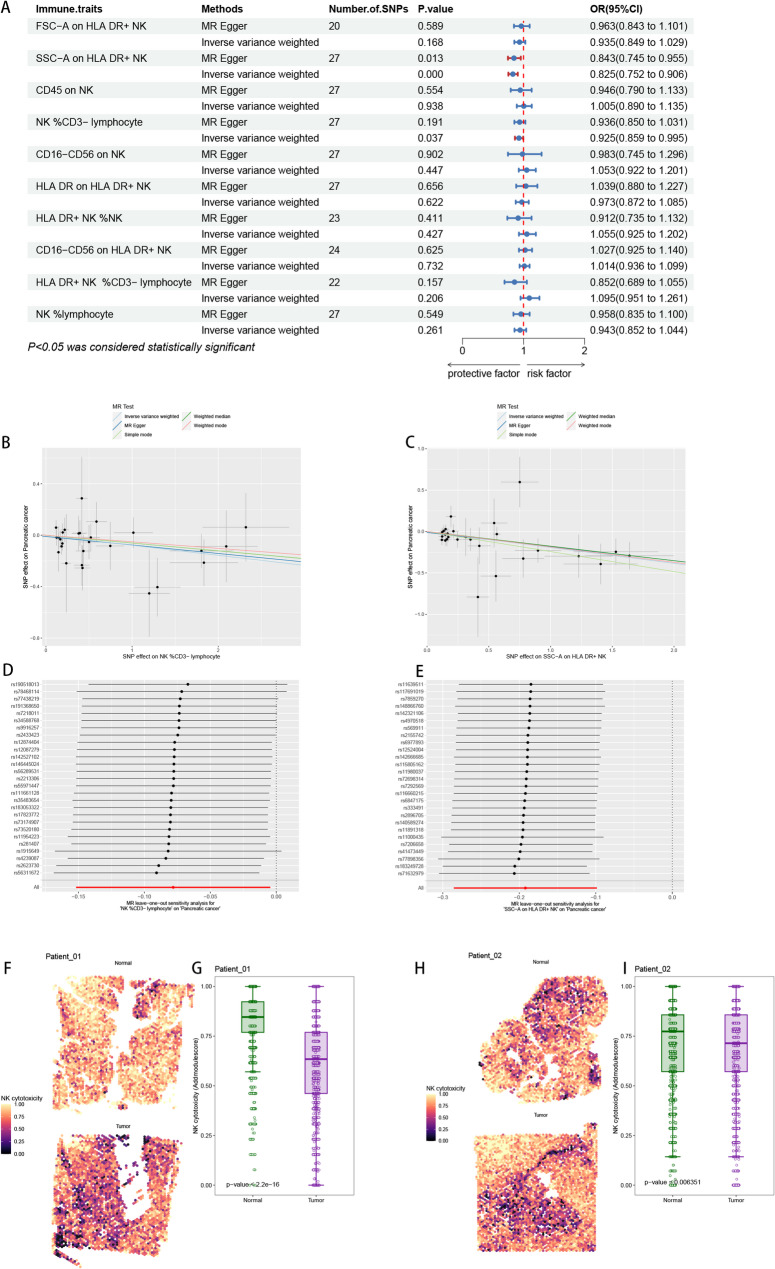
Mendelian randomization and spatial transcriptomic analyses reveal impaired cytotoxic function of NK cells in pancreatic cancer. (**A**) Causal associations between NK-cells related immune traits and pancreatic cancer with mendelian randomization. SNP, single nucleotide polymorphism; OR, odds ratio; CI, confidence interval. HLA, human leucocyte antigen; NK, natural killer cells. (**B**) Scatterplot of SNP effects on NK% CD3- lymphocyte versus pancreatic cancer. (**C**) Scatterplot of SNP (single nucleotide polymorphism) effects on SSC-A on HLA DR+ NK (natural killer) cells versus pancreatic cancer.(**D**) The leave-one-out sensitivity analysis for NK% CD3- lymphocyte versus pancreatic cancer. (**E**) The leave-one-out sensitivity analysis for SSC-A on HLA DR+ NK cells versus pancreatic cancer.(**F**–**G**) Spatial transcriptomic analysis of Patient_01 showing significantly lower NK cytotoxicity scores in tumor regions compared with paired normal regions (*p* < 2.2 × 10^− 1 6^). (**H**–**I**) Similar results were observed in Patient_02, with markedly reduced NK cytotoxicity in tumor tissues (*p* = 0.006351)

To investigate the causal relationship between NK-cell related immune characteristics and pancreatic cancer risk, we performed Mendelian randomization (MR) analysis on ten NK-cell associated phenotypes (Fig. 1A). Among these, the morphological parameter SSC-A on HLA DR^+^ NK cells showed a significance inverse association with pancreatic cancer risk in both the inverse-variance weighted (IVW) method (OR = 0.825, 95% CI = 0.752–0.906, *p* < 0.0001) and MR-Egger regression analysis (OR = 0.843, 95% CI = 0.745–0.955, *p* = 0.013). This finding suggests that reduced NK cell granularity or activation status, as reflected by SSC-A, may increase susceptibility to pancreatic cancer. Additionally, in the IVW analysis, a higher NK cell/CD3^−^ lymphocyte ratio was negatively associated with pancreatic cancer risk (OR = 0.925, 95% CI = 0.859–0.995, *p* = 0.037), but this association did not reach significance in the MR-Egger model (OR = 0.936, 95% CI = 0.850–1.031, *p* = 0.191) (Fig. 1B–C). Sensitivity analyses revealed no evidence of heterogeneity (SSC-A on HLA DR^+^ NK, *p* = 0.679; NK cell/CD3^−^ lymphocyte ratio, *p* = 0.939) or horizontal pleiotropy (SSC-A on HLA DR^+^ NK, *p* = 0.609; NK cell/CD3^−^ lymphocyte ratio, *p* = 0.706), confirming the robustness of causal estimates. The leave-one-SNP-out analysis further validated the absence of any single variant substantially influencing the overall causal effect (Fig. 1D–E). Although both NK-cell related features showed negative correlations with pancreatic cancer risk, only SSC-A on HLA DR^+^ NK cells, a proxy for NK-cell activation and cytotoxic potential, remained significant in both MR models. This pattern suggests that functional impairment rather than numerical reduction of NK cells may present the key immune alteration driving susceptibility to pancreatic cancer. Thus, our subsequent validation analyses focused specifically on the cytotoxic function of NK cells.

To validate these genetic findings at the tissue level, we further assessed NK-cell cytotoxic activity using spatial transcriptomic data from paired pancreatic tumor and normal tissues. Results showed that cytotoxicity scores for NK cells in tumor regions were markedly reduced than those in normal controls in both Patient_01 (*p* < 2.2 × 10^− 1 6^; Fig. 1F–G) and Patient_02 (*p* = 0.006351; Fig. 1H–I). Collectively, these findings suggested that functional exhaustion of NK cells, characterized by decreased cytotoxic potential, may represent a key immunologic mechanism promoting pancreatic cancer progression.

To further elucidate the clinical significance of NK cell dysfunction in pancreatic cancer, we constructed a prognostic model based on NK-cell specific genes using single-cell RNA sequencing data (Supplementary Fig. 1A–D). Patients were stratified into high-risk and low-risk groups according to risk score derived from the LASSO algorithm. In both the TCGA and GEO cohorts, the high-risk group exhibited significantly poorer overall survival compared with the low-risk group (Supplementary Fig. 1E). Subsequent functional enrichment analyses revealed the biological pathways associated with the risk model. GO analysis indicated significantly enrichment of risk-related genes in immune processes, including regulation of immune effector process, activation of immune response, and immune response–regulating cell surface receptor signaling pathway. KEGG analysis similarly highlighted pathways such as cytokine–cytokine receptor interaction, cell adhesion molecules (CAMs), and primary immunodeficiency (Supplementary Fig. 1 G).

Notably, GSEA analysis demonstrated a pronounced downregulation of the natural killer cell–mediated cytotoxicity pathway in the high-risk group (Supplementary Fig. 1 H), consistent with our spatial transcriptomic findings that revealed reduced NK-cell cytotoxic activity in the pancreatic tumor microenvironment. These results collectively indicated that impaired NK cell cytotoxic function represents a key immunologic mechanism contributing to poor prognosis in pancreatic cancer.

### Identification of a tumor-associated NK-cell subset with reduced cytotoxicity and poor prognosis in PDAC

**Fig. 2:**
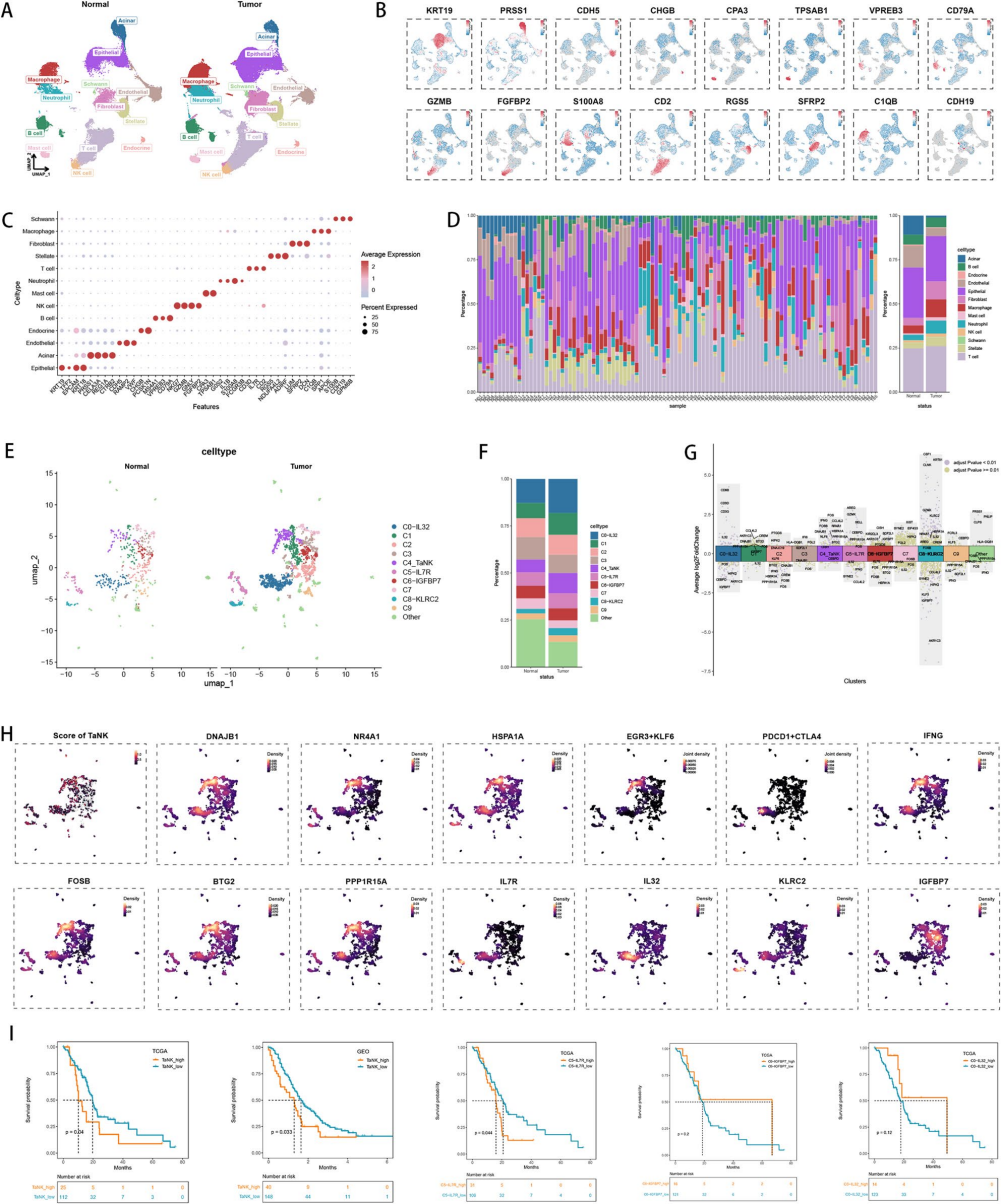
Identification of tumor-associated NK cell subtypes reveals a TaNK population linked to poor prognosis in pancreatic cancer. (**A**) UMAP visualization of 51,673 cells from adjacent normal tissues and 143,858 cells from tumor tissues of 102 PDAC patients, revealing 13 major cell clusters. Batch effects were corrected using the Harmony package, and all cells were jointly embedded, then separated by tissue origin. (**B**) Expression patterns of representative marker genes across 195,531 cells, visualized on the UMAP plots for both normal and tumor tissues. (**C**) Dot plot showing the average expression and detection frequency of canonical marker genes across identified cell clusters. Dot size indicates the proportion of expressing cells, and color intensity represents expression level. (**D**) Distribution of 13 major cell types across individual donors (left) and between normal and tumor tissues (right). PDAC, pancreatic ductal adenocarcinoma. (**E**) UMAP visualization of tumor-infiltrating NK cells colored by cell subtype. (**F**) Stacked bar plot showing NK cell composition across different tissue types. (**G**) Differentially expressed genes among NK cell subsets. (**H**) TaNK scores calculated using the AddModuleScore function, with corresponding marker gene expression shown on the UMAP plot. (**I**) High infiltration of TaNK and C5–IL7R NK cells is associated with poorer prognosis, whereas other NK subtypes show no significant correlation with survival

To comprehensively understand the heterogeneity of NK cells in PDAC, we integrated previously published single-cell datasets, encompassing 102 single-cell samples from both tumor and normal tissues [13, 25, 26]. Subsequent analysis included a total of 195,531 cells, comprising 143,858 cells from tumor tissues and 51,673 cells from adjacent normal tissues (Fig. 2A) [27, 28]. Based on established gene markers, we identified 13 cell types (Fig. 2B, C): Fibroblasts (*n* = 16,761) exhibited high expression of LUM, SFRP2, and DCN; Mast cells (*n* = 3,206) were marked by TPSAB1 and CPA3; Macrophages (*n* = 17,013) were identified with C1QB, SPP1, and APOE; Neutrophils (*n* = 12,202) expressed G0S2 and FCGR3B; Schwann cells (*n* = 409) were characterized by high CDH19 expression; Epithelial cells (*n* = 51,679) were identified by high expression of KRT19 and EPCAM; Endothelial cells (*n* = 12,939) expressed VWF and CDH5; Acinar cells (*n* = 7,039) were identified by PRSS1 and CELA3A; B cells (*n* = 10,983) were marked by MS4A1; Cells with high expression of CHGB and PCSK1N were considered endocrine cells (*n* = 884); NK cells (*n* = 3,443) were identified by high expression of NKG7, GZMB, and FGFBP2; T cells (*n* = 50,340) highly expressed CD3D and CD2; Stellate cells (*n* = 8,759) were identified by RGS5 and ADIRF. The infiltration levels of these 13 cell types varied across different tissues. Notably, although NK cells were more abundant in tumor tissues than in normal tissues (Fig. 2D), their functional status in the TME remained unclear. Given the immunosuppressive nature of pancreatic cancer, we next analyzed NK cell subsets to determine which may exhibit diminished cytotoxic activity.

Recent pan-cancer analyses have identified a TaNK subset characterized by reduced cytotoxicity and an immunoregulatory phenotype [29]. However, it remains unclear whether such NK-cell subsets function similarly within the highly fibrotic and immunosuppressive microenvironment of PDAC. To address this, we integrated large-scale single-cell RNA sequencing data from PDAC and adjacent normal tissues, systematically annotating NK cell subsets based on established marker genes (Fig. 2E, H). Through unsupervised clustering combined with TaNK-specific gene scoring, we identified Cluster 4 (C4-TaNK; *n* = 347) as a distinct tumor-associated NK subset. This population exhibited high DNAJB1 expression and upregulation of NR4A1 and HSPA1A, consistent with features of previously reported TaNK cells. C4-TaNK also overexpressed KLF6 and EGR3 (transcription factors known to suppress NK cytotoxicity), while showing negligible expression of classic immune checkpoint genes (PDCD1, CTLA4)—further confirming its identity as a functionally impaired, tumor-associated NK subset. Additional NK subtypes were identified, including C0–IL32, C5–IL7R, C6–IGFBP7, and C8–KLRC2, each defined by charasteristic genes (Fig. 2H). Notably, we also identified IFNG, FOSB, BTG2, and PPP1R15A as novel markers associated with TaNK cells. Cross-tissue analysis revealed significantly higher abundance of TaNK cells in tumor regions compare to normal pancreas (Fig. 2F), with the differential expression patterns shown in Fig. 2G.

Clinically, patients with high TaNK infiltration exhibited significantly poorer overall survival in the TCGA cohort (*p* = 0.040), with results validated in an independent GEO dataset (*p* = 0.033) (Fig. 2I). We also observed that the C5–IL7R NK-cell subset was associated with worse prognosis (*p* = 0.044), suggesting its potential immunoregulatory role in PDAC. In contrast, other NK subsets show no significant correlation with prognosis. To summarize, these findings suggested that tumor-resident TaNK cells constitute a dysfunctional NK-cell subset with impaired cytotoxic activity, potentially promoting PDAC progression and leading to poor clinical outcomes. We therefore hypothesize that specific stromal or immune cell populations within the TME of PDAC may promote the differentiation or maintenance of these cytotoxic-impaired TaNK cells through unique intercellular signaling mechanisms [30].

### myCAF subtype exhibits the strongest interaction with TaNK cells and correlates with poor prognosis in PDAC

**Fig. 3:**
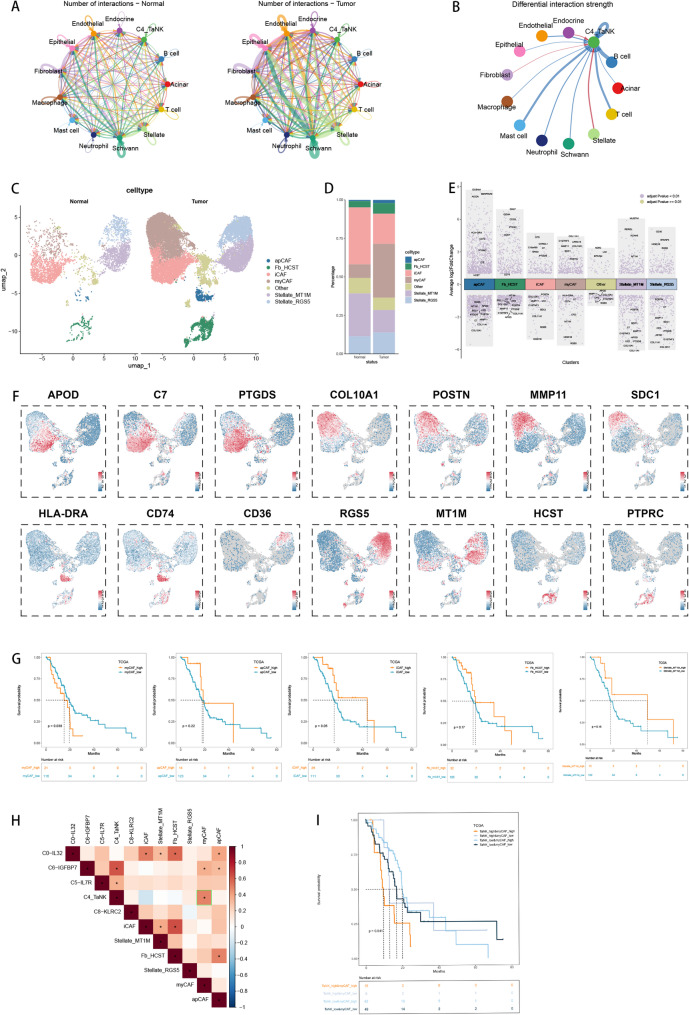
Crosstalk between fibroblast subtypes and TaNK reveals myCAF–TaNK interaction associated with poor prognosis in pancreatic cancer. (**A**) Network analysis showing that multiple cell types actively interact with TaNK cells within the TME. (**B**) Among all interacting cell types, fibroblasts and stellate cells exhibit the strongest communication with TaNK cells. (**C**) UMAP visualization of tumor-infiltrating fibroblasts colored by cell subtype. (**D**) Stacked bar plot illustrating fibroblast composition across normal and tumor tissues. (**E**) Differentially expressed genes among fibroblast subpopulations. (**F**) UMAP plots showing the expression of representative marker genes in fibroblasts from normal and tumor tissues of PDAC. (**G**) High infiltration of myCAF cells is associated with poor prognosis, while other fibroblast subsets show no significant correlation with survival. (**H**) Correlation analysis revealing a significant positive association between myCAF and TaNK cell infiltration. (**I**) Combined survival analysis demonstrating that patients with high infiltration of both myCAF and TaNK cells have the worst prognosis

To identify regulators that may influence TaNK function in PDAC, we used CellChat to analyze intercellular communication networks within the TME. TaNK cells displayed extensive crosstalk with multiple cell types (Fig. 3A). Notably, both fibroblasts and stellate cells exhibited markedly enhanced interaction strength with TaNK cells in tumor tissues, suggesting their crucial role in regulating NK cell functions (Fig. 3B). Thus, we focused our subsequent analyses on fibroblasts and pancreatic stellate cells within the TME of PDAC. Subsequently, we extracted fibroblasts and pancreatic stellate cells from the integrated single-cell dataset and performed unsupervised clustering to define their subpopulations (Fig. 3C, F) [31]. This analysis identified six transcriptionally distinct clusters, whose differential gene expression patterns are illustrated in Fig. 3E. Among the stellate cells, two subsets were identified: Stellate_MT1M (*n* = 4,229; characterized by MT1M expression) and Stellate_RGS5 (*n* = 3,452; expressing RGS5 and CD36). The fibroblast compartment was further divided into four clusters. In agreement with previous reports [32], we annotated myCAF (*n* = 7,915; COL10A1, POSTN, MMP11, SDC1 positive), apCAF (*n* = 498; HLA-DRA, CD74 positive), and iCAF (*n* = 5,661; APOD, C7, PTGDS positive). A minor group labeled as “Other” lacked clear marker genes. In addition, we discovered a previously uncharacterized fibroblast subcluster defined by high expression of HCST and PTPRC, designated Fb_HCST (*n* = 1,640). This novel subtype may represent a functionally distinct stromal population in PDAC, meriting further investigation.

By comparing the infiltration levels of fibroblast subtypes across tumor and normal tissues, we found that myCAF cells were markedly enriched in tumor regions compared to normal pancreatic tissues (Fig. 3D). To assess their clinical relevance, we evaluated the association between fibroblast subtype infiltration and patient survival in PDAC. In the TCGA cohort, high myCAF infiltration was significantly associated with poorer overall survival, whereas the infiltration levels of other fibroblast and stellate subtypes showed no notable prognostic impact (Fig. 3G). Given these findings, we hypothesized that myCAFs within the TME of PDAC might influence the biological properties of tumor-resident TaNK cells. Indeed, correlation analysis revealed a strong positive association between myCAF and TaNK infiltration levels in PDAC tissues (Fig. 3H). To further elucidate the clinical significance of this relationship, we compared survival outcomes according to combined infiltration status. Patients with concurrent high infiltration of both myCAF and TaNK cells exhibited the poorest overall survival, suggesting that these two cell populations may cooperatively promote tumor progression (Fig. 3I, *p* = 0.041).

### Spatial transcriptomics reveals co-localization of myCAF and TaNK cells and links myCAF abundance to reduced NK-cell cytotoxicity in PDAC

**Fig. 4:**
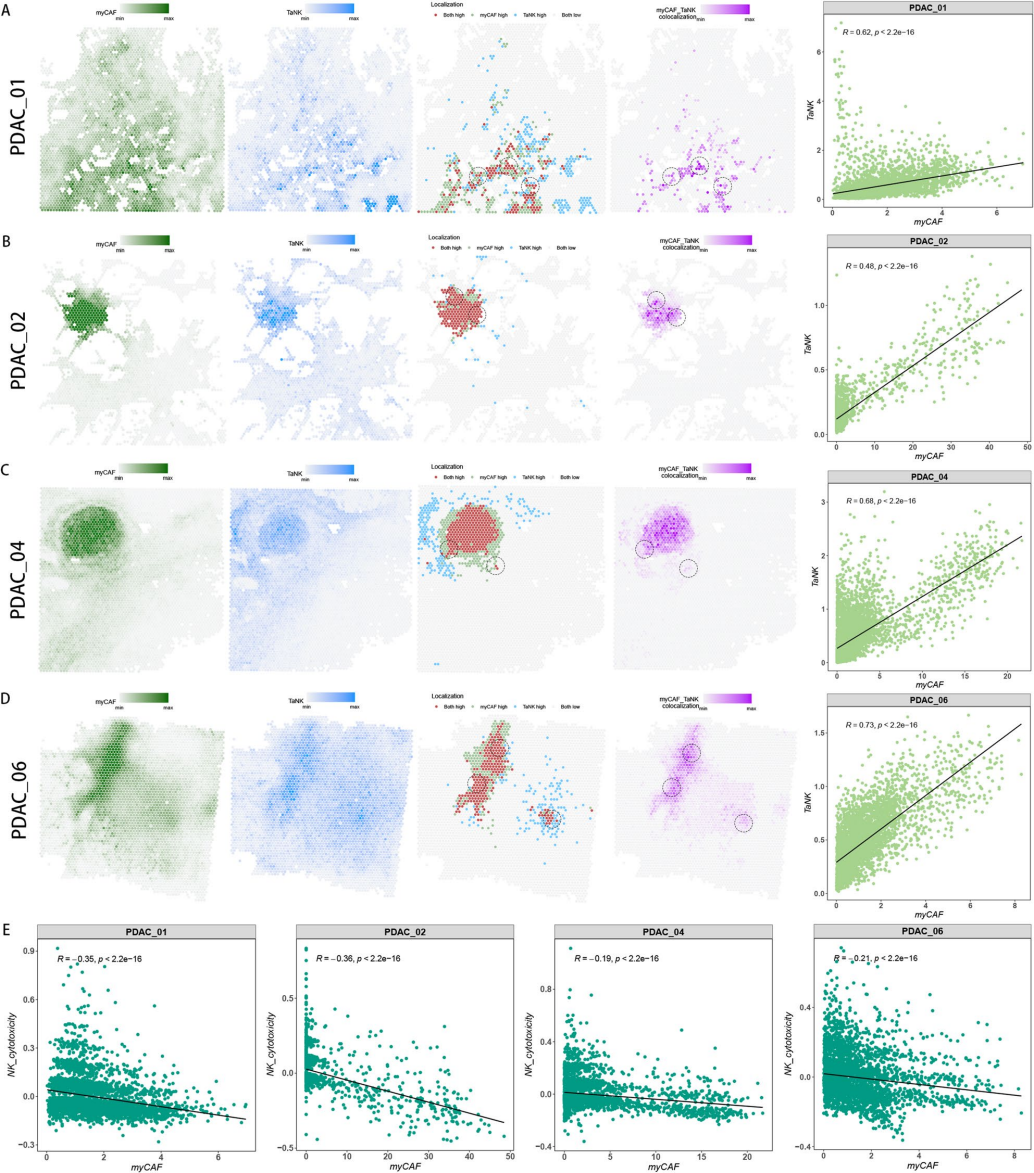
Spatial co-localization of myCAF and TaNK cells reveals myCAF-associated suppression of NK cell cytotoxicity in pancreatic cancer. (A–D) Spatial mapping of myCAF and TaNK cells across four PDAC samples (PDAC_01, PDAC_02, PDAC_04, and PDAC_06). From left to right: spatial distribution of myCAF cells, spatial distribution of TaNK cells, spatial localization of both cell types, co-localization maps highlighting representative overlapping regions (indicated by black dashed circles), and correlation analyses showing strong positive spatial associations between myCAF and TaNK distributions in all samples (PDAC_01: R = 0.62; PDAC_02: R = 0.48; PDAC_04: R =0.68; PDAC_06: R =0.73; all p < 2.2e–16). (E) Correlation analyses showing the spatial relationship between myCAF distribution and NK cell cytotoxicity across four PDAC samples. All samples exhibit significant inverse correlations (PDAC_01: R = −0.35; PDAC_02: R = −0.36; PDAC_04: R = −0.19; PDAC_06: R = −0.21; all p < 2.2e–16)

Single-cell transcriptomic analysis revealed strong interactions between myCAFs and TaNK cells within the PDAC microenvironment. However, the spatial organization patterns of these two populations could not be inferred from single-cell data alone. To address this, we investigated their spatial relationships through spatial transcriptomic colocalization analysis. Across multiple PDAC samples, myCAFs and TaNK cells exhibited prominent spatial co-localization, with significant positive correlations observed in all four representative specimens (PDAC_01: *R* = 0.62; PDAC_02: *R* = 0.48 ; PDAC_04: *R* = 0.68 ; PDAC_06: *R* = 0.73 ; all *p* < 2.2 × 10^− 1 6^) (Fig. 4A–D). Based on these findings, we hypothesized that spatial proximity to myCAFs may impair NK-cell cytotoxicity. Correlation analyses confirmed a significant inverse association between myCAF abundance and NK-cell cytotoxic activity (PDAC_01: *R* = −0.35; PDAC_02: *R* = −0.36; PDAC_04: *R* = −0.19; PDAC_06: *R* = −0.21; all *p* < 2.2 × 10^− 1 6^) (Fig. 4E). Reproducible results obtained across four independent sections further confirmed that myCAFs spatially associated with NK cells in the PDAC microenvironment and impaired their cytotoxic activity.

### myCAFs regulate TaNK cells through collagen signaling, validated by spatial co-localization of cells and ligand–receptor pairs

**Fig. 5:**
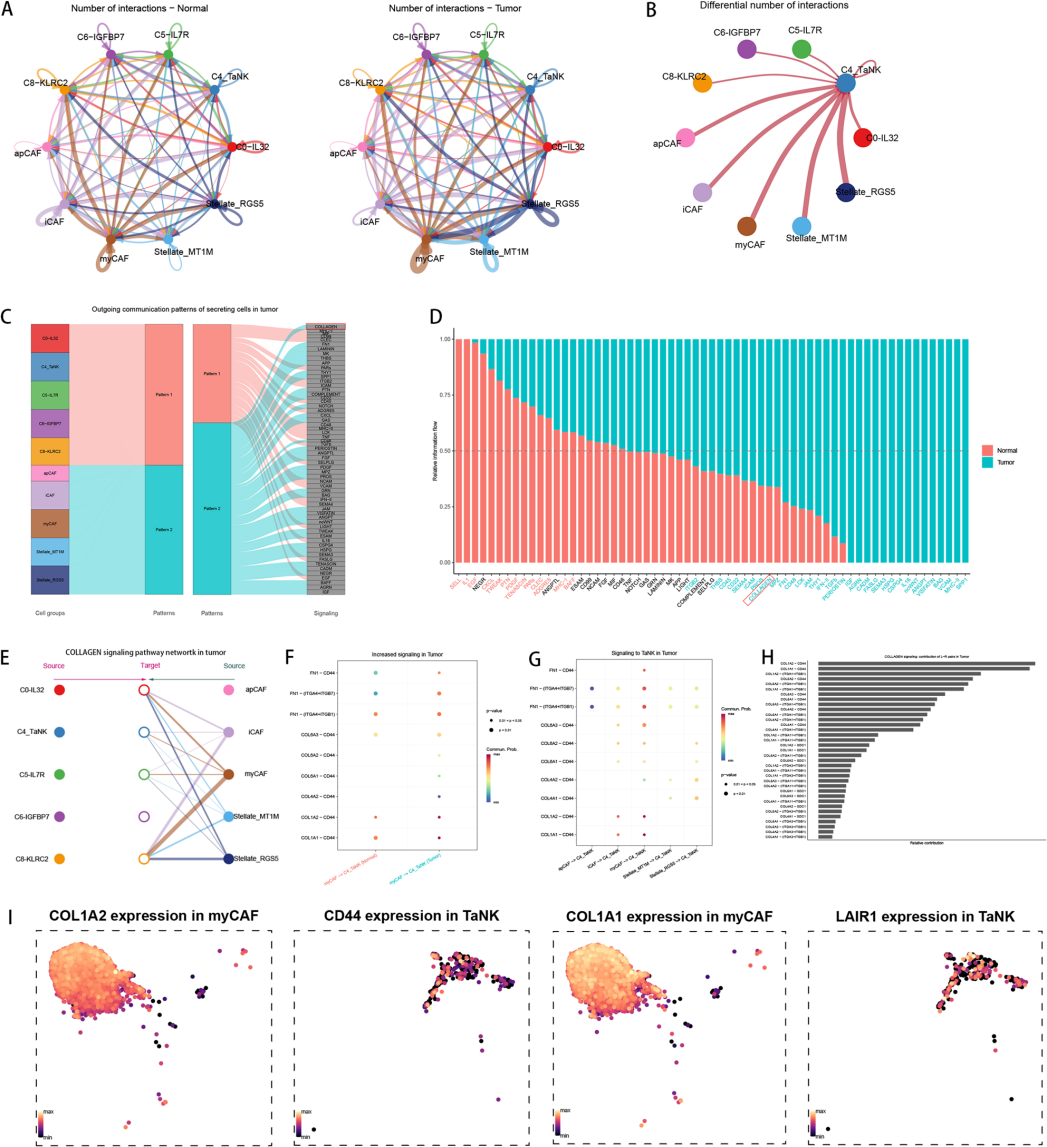
Identification of tumor-specific signaling pathways in PDAC based on fibroblast–NK cell regulatory network (**A**). Circle plots showing the global intercellular communication network between fibroblast and NK-cell subsets in normal (left) and tumor (right) tissues. The width of the connecting lines indicates the number of interactions; thicker lines represent stronger communication. (**B**). Differential interaction network highlighting enhanced communication between myCAF and TaNK cells in tumor tissue. (**C**). River plot illustrating the outgoing communication patterns of fibroblast subsets in PDAC tumor tissue. (**D**). Bar plot displaying signaling pathways with tumor- or normal-specific enrichment between fibroblasts and NK cells, as inferred by CellChat. Red bars indicate pathways enriched in normal tissue, whereas blue bars indicate pathways enriched in tumor tissue. (**E**). Hierarchical network visualization of the COLLEGEN signaling pathway, showing myCAF-mediated regulation of TaNK cells in tumor tissue. (**F**–**G**) dot plots showing tumor-specific enhancement of FN1 and COLLAGEN signaling pathways between myCAF (source) and TaNK (target) cells in PDAC tissue. (**H**). Bar plot showing the contribution of all LR pairs within the collagen pathway in tumor tissue. (**I**). Expression level of COLA2 and COL1A1 in myCAF, CD44, LAIR1 in TaNK

Extensive research indicated that interactions between fibroblasts and NK cells profoundly influence tumor progression and immune regulation [33]. Our spatial transcriptomics analysis revealed close spatial colocalization between myCAFs and TaNK cells, along with a potential inhibitory effect of myCAFs on NK-cell cytotoxicity. However, the molecular basis and signaling pathways underlying this interaction remained unclear. To further elucidate the intercellular communication mechanisms between these populations, we applied CellChat to construct comprehensive ligand–receptor interaction networks between fibroblast subtypes and NK-cell subtypes in tumor and normal pancreatic tissues. Results demonstrated significantly increased intercellular communication complexity in tumor tissues compared to normal tissues (Fig. 5A). In-depth analysis of fibroblast and stellate cell subtypes revealed significantly elevated interaction frequencies with TaNK cells in tumor tissue (Fig. 5B). Given that myCAFs served as the primary stromal cells responsible for extracellular matrix (ECM) production in PDAC, we hypothesized that myCAFs may regulate NK-cell function primarily through ECM-related signaling pathways.

To validate this hypothesis, we performed outgoing communication pattern analysis using CellChat. As shown in Fig. 5C, collagen signaling emerged as one of the dominant fibroblast-derived outgoing pathways in tumor tissues, suggesting its potential involvement in fibroblast–immune cell crosstalk. Consistently, the relative information flow of the collagen pathway was markedly elevated in tumors compared with normal tissues (Fig. 5D), indicating that collagen-mediated intercellular signaling was specifically enhanced in tumors. In-depth analysis of the collagen signaling network revealed extensive collagen-based communication between multiple fibroblast and stellate cell subtypes and NK-cell populations (Fig. 5E). The study further discovered that myCAFs regulate TaNK cells via the collagen signaling pathway, providing additional evidence for the potential functional association between these two cell types in the PDAC microenvironment. To elucidate the molecular basis of collagen-mediated communication, we systematically examined the ligand–receptor pairs connecting myCAFs and TaNK cells. As shown in Fig. 5F–G, compared to normal controls, both COL1A1–CD44 and COL1A2–CD44 interactions were significantly upregulated in tumor tissues, indicating enhanced collagen–receptor binding in PDAC. Quantitative contribution analysis revealed that the COL1A2–CD44 pair exhibited the highest contribution score within the collagen signaling network (Fig. 5H), identifying it as a key molecular bridge mediating myCAF–TaNK communication in PDAC. Subsequently, we examined the expression of these key ligand–receptor pairs at the single-cell level. COL1A2 was highly expressed in myCAFs, while its corresponding receptor CD44 was predominantly expressed in TaNK cells, suggesting potential communication between these cell types via the collagen signaling pathway (Fig. 5I). Recent studies have reported that fibroblasts can negatively regulate NK-cell cytotoxicity through the COL1A1–LAIR1 ligand–receptor interaction [34, 35]. Consistently, the single-cell data showed COL1A1 expression in myCAFs and detectable LAIR1 in TaNK cells, indicating an additional potential inhibitory axis between these two cell types.

**Fig. 6:**
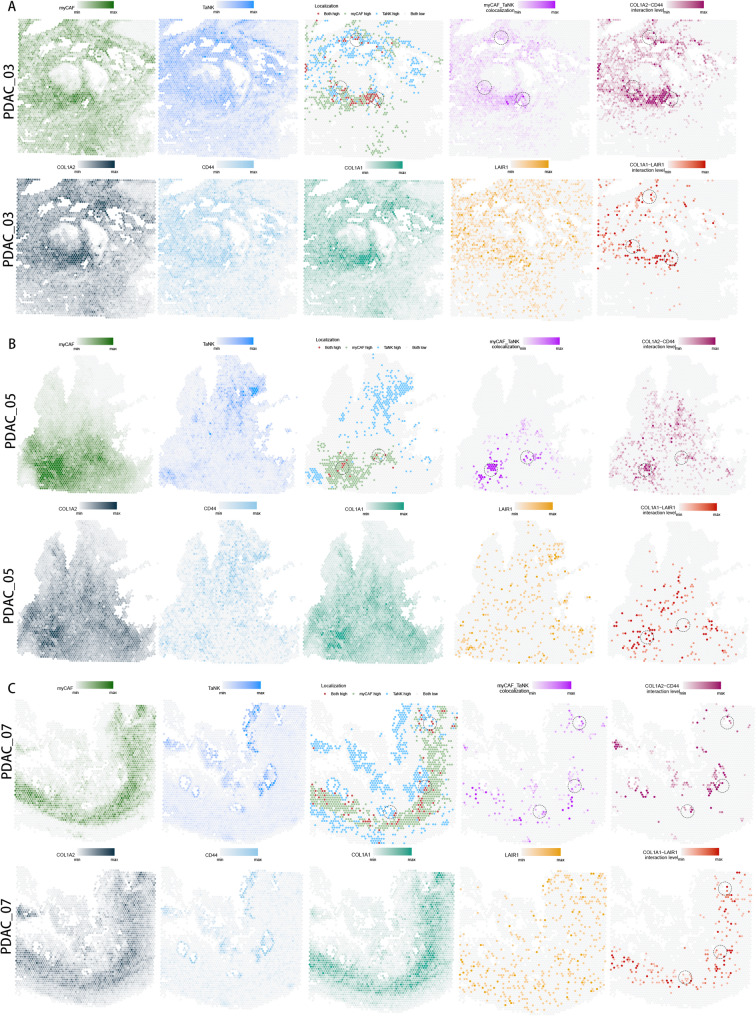
Spatial colocalization of myCAF–TaNK cells and COL1A2–CD44 / COL1A1–LAIR1 ligand–receptor pairs in PDAC. (**A**) Representative spatial transcriptomic maps of sample PDAC_03. From left to right (top row): spatial distribution of myCAF cells, TaNK cells, their spatial localization map, colocalization intensity between myCAF and TaNK, and spatial interaction level of the COL1A2–CD44 ligand–receptor pair. From left to right (bottom row): spatial expression of COL1A2, CD44, COL1A1, and LAIR1, as well as the spatial interaction level of the COL1A1–LAIR1 ligand–receptor pair. Dashed circles indicate representative regions where myCAF and TaNK cells exhibit close spatial proximity and elevated ligand–receptor interaction strength. (**B**–**C**) Spatial maps of PDAC_05 and PDAC_07, respectively, showing consistent spatial patterns of myCAF and TaNK colocalization, along with elevated COL1A2–CD44 and COL1A1–LAIR1 interaction signals, similar to those observed in PDAC_03

To further validate these findings at the spatial level, we performed colocalization analysis using spatial transcriptomics data from PDAC tissues. In PDAC_03 (Fig. 6A), myCAFs and TaNK cells exhibited clear spatial colocalization, and notably, the COL1A2–CD44 ligand–receptor pair also showed strong spatial overlap. Additionally, COL1A1–LAIR1 exhibited a similar colocalization pattern, suggesting potential involvement of both collagen-mediated signaling axes in the TME. Consistent results were obtained in PDAC_05 (Fig. 6B) and PDAC_07 (Fig. 6C), supporting the robustness and reproducibility of these spatial interaction patterns.

### Multiplex immunofluorescence validates spatial co-localization of myCAFs and NK cells and their association with reduced NK-cell cytotoxicity in PDAC

**Fig. 7:**
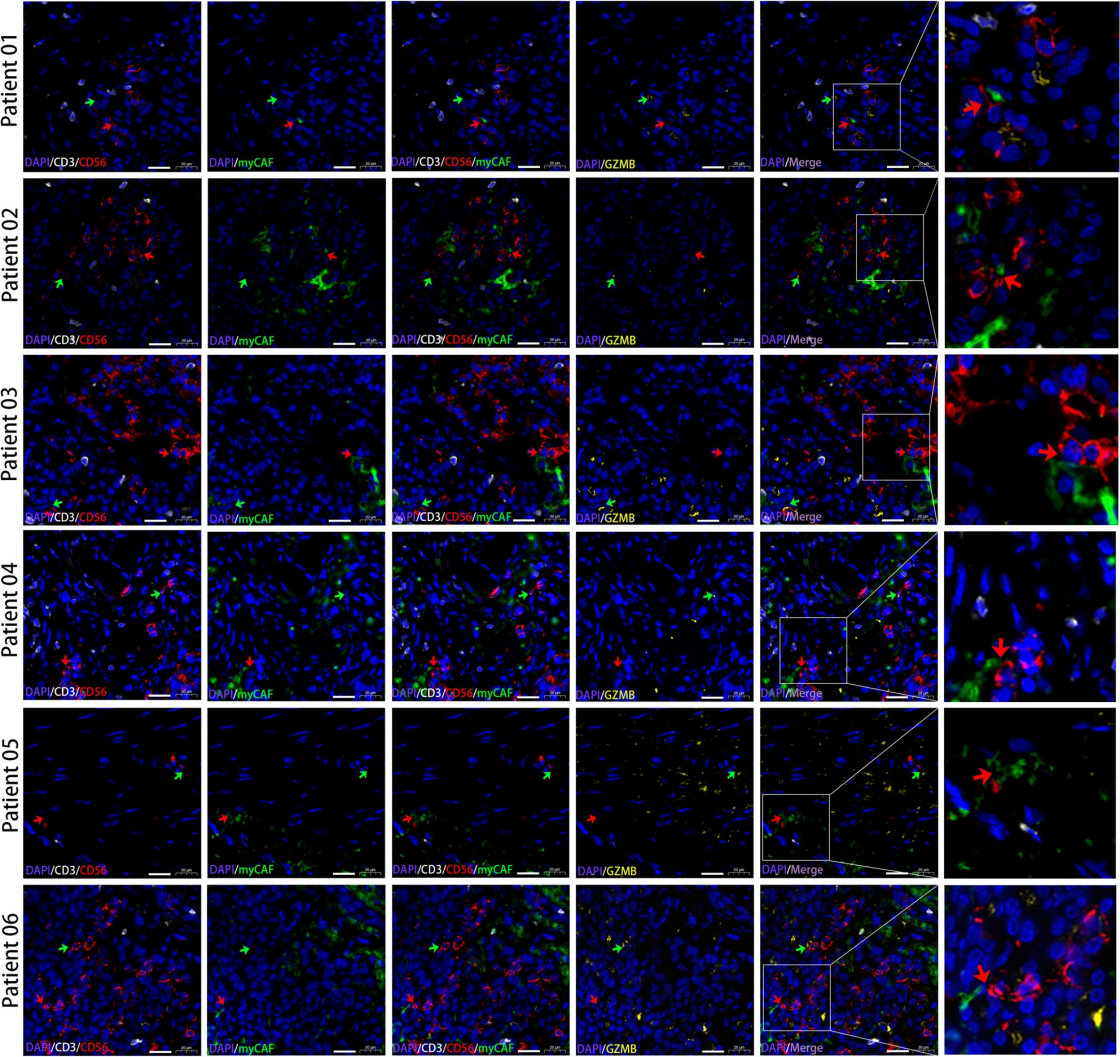
Multiplex immunofluorescence staining to show the colocalization of myCAF (POSTN+) and NK cells (CD3–CD56+). Scale bar = 20 μm (40× objective)

To further validate, at the tissue level, the spatial proximity between myCAFs and NK cells and the potential suppressive effect of myCAFs on NK cell cytotoxicity, we performed multiplex immunofluorescence staining on PDAC specimens. CD3^−^ CD56^+^ was used to identify NK cells, POSTN^+^ as a marker for myCAFs, and GZMB to assess NK cell cytotoxic activity. In line with the spatial transcriptomic findings, myCAFs and NK cells were frequently colocalized in the TME. Notably, NK cells adjacent to POSTN^+^ myCAFs exhibited markedly lower GZMB expression, indicating diminished cytotoxic potential. This pattern was reproducibly observed across six independent PDAC samples, emphasizing the high reliability and biological relevance of myCAF-mediated NK cell suppression (Fig. 7).

### Co-enrichment of myCAFs and TaNK cells predicts an immune-excluded microenvironment and immunotherapy resistance in PDAC

**Fig. 8:**
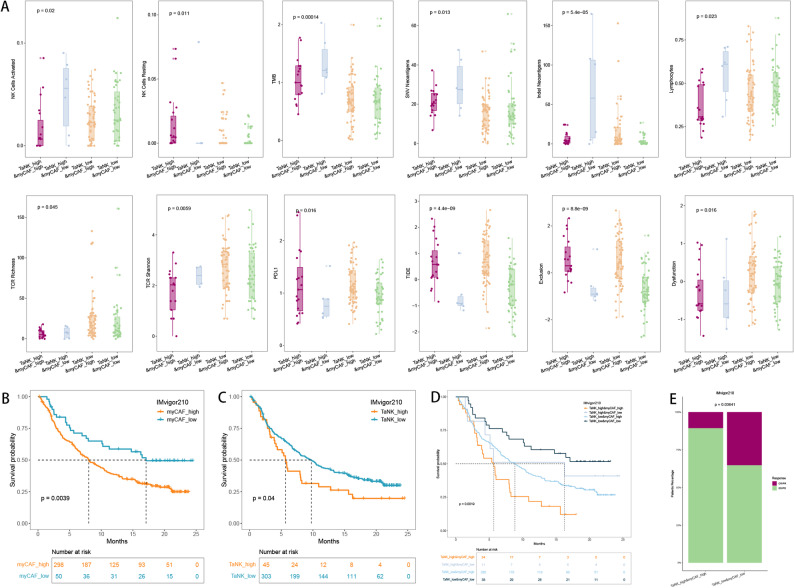
Impact of myCAF and TaNK cell infiltration on the immunological and clinical outcomes of patients undergoing immunotherapy. (A) comparison of immune-related molecular and cellular features among four TCGA PDAC subgroups stratified by myCAF and TaNK cell infiltration levels. From left to right (top row), boxplots display differences in activated NK cells, resting NK cells, tumor mutation burden (TMB), SNV neoantigens, indel neoantigens, and lymphocyte infiltration; and TCR richness, TCR Shannon index, PD-L1 expression, TIDE score, T-cell exclusion, and T-cell dysfunction (bottom row). Statistical differences were assessed using one-way ANOVA. (B-D) Kaplan–Meier survival analyses of the IMvigor210 anti–PD-L1 immunotherapy cohort, comparing patients stratified by myCAF infiltration (B), TaNK infiltration (C), and the combined classification of myCAF and TaNK infiltration levels (D). Survival differences were evaluated using a two-sided log-rank test. (E) Bar plot showing the proportion of patients with clinical response (CR/PR) and non-response (SD/PD) in each subgroup. Patients with concurrent high infiltration of myCAF and TaNK cells exhibited the poorest response to anti–PD-L1 treatment. Statistical significance was evaluated using Fisher’s exact test. Abbreviations: PDAC, pancreatic ductal adenocarcinoma; TCGA, the cancer genome Atlas; SNV, single-nucleotide variant; TCR, T-cell receptor; TIDE, tumor immune dysfunction and exclusion. CR/PR: complete response/partial response; SD/PD: stable disease/progressive disease

To further delineate the immunological characteristics of PDAC tumors co-enriched with myCAFs and TaNK cells, patients were stratified into four groups based on the infiltration levels of myCAFs and TaNKs. As shown in Fig. 8A, tumors exhibiting high infiltration of both myCAFs and TaNK cells displayed distinct immunological profile characterized by reduced NK-cell activation (*p* = 0.02) and increased resting NK-cell proportions (*p* = 0.011), indicative of suppressed innate cytotoxic activity. Despite exhibiting elevated tumor mutation burden (TMB, *p* = 0.00014) and higher neoantigen loads derived from both SNVs (*p* = 0.013) and indels (*p* = 5.4 × 10^− 5^), they paradoxically showed reduced lymphocyte infiltration (*p* = 0.023), suggesting an immune exclusion microenvironment despite high immunogenic potential. Consistently, the TCR richness (*p* = 0.045) and Shannon diversity index (*p* = 0.0059) were significantly decreased in the dual-high group, reflecting a more clonal and less diverse adaptive immune repertoire. Analysis of immune checkpoint and evasion-related parameters further revealed moderately elevated PD-L1 expression (*p* = 0.016), accompanied by significantly increased TIDE (*p* = 4.4 × 10^− 9^) and Exclusion scores (*p* = 8.8 × 10^− 9^), along with markable reduction in Dysfunction score (*p* = 0.016). This pattern, characterized by strong immune exclusion but diminished immune dysfunction, aligns with the classical immune-excluded phenotype, where dense stromal barriers restrict immune-cell infiltration into the tumor parenchyma, thereby preventing effective anti-tumor immune activation [36].

To further evaluate the clinical significance of the myCAF–TaNK signature, we analyzed patient outcomes in the IMvigor210 cohort. This cohort represented a well-characterized dataset of urothelial carcinoma patients treated with anti–PD-L1 therapy. As shown in Fig. 8B, patients with high myCAF infiltration exhibited significantly shorter overall survival compared to those with low myCAF levels (*p* = 0.0039). Similarly, high TaNK infiltration was also associated with poorer prognosis (*p* = 0.040, Fig. 8C). When both parameters were considered concurrently, patients exhibiting both high myCAF and high TaNK infiltration demonstrated the lowest overall survival across all subgroups (*p* = 0.0019, Fig. 8D). This further substantiated the notion that fibroblast-mediated stromal remodeling and NK cell dysfunction synergistically promote immune evasion and disease progression. Patients were classified into responders (CR/PR) and non-responders (SD/PD) based on their best response to anti–PD-L1 therapy. As shown in Fig. 8E, the proportion of responders (CR/PR) in the dual-high group was markedly lower than that in the dual-low group (*p* = 0.03641), whereas the non-responder population (SD/PD) predominated in tumors co-enriched with myCAFs and TaNK cells. These findings indicated that PDAC tumors co-enriched with myCAFs and TaNK cells exhibit reduced sensitivity to PD-L1 blockade, likely driven by an immune-excluded, stromal-dominant microenvironment that impedes cytotoxic lymphocyte infiltration and activation.

## Discussion

In this study, we comprehensively explored the heterogeneity of NK cell and its underlying stromal regulation within the TME of PDAC. Mendelian randomization analysis revealed that impaired NK cell function, rather than decreased cell numbers, correlated with elevated PDAC risk. Spatial transcriptomic analysis further confirmed markedly reduced NK cell cytotoxicity in tumor tissues compared to matched normal pancreas. Integrating single-cell and spatial analyses identified a TaNK exhibiting diminished cytotoxicity and close association with myCAFs. CellChat analysis revealed collagen-related communication between these two cell types, primarily through COL1A2–CD44 and COL1A1–LAIR1 ligand–receptor pairs. Previous reports indicated that COL1A2–CD44 signaling limited NK-cell adhesion and migration, while COL1A1–LAIR1 interactions suppressed cytotoxic activity, collectively leading to NK cell dysfunction [34, 37, 38]. Spatial and multiplex immunofluorescence analyses validated the spatial colocalization of myCAFs and TaNKs. Tumors enriched with both celltypes exhibited an immune-excluded phenotype and reduced sensitivity to PD-L1 blockade therapy. These findings elucidated a stromal–immune axis in which collagen signaling from myCAFs induced NK-cell dysfunction and immunotherapy resistance in PDAC.

Recent studies have shown that specific immune populations, such as LAMP3^+^ dendritic cells, could suppress NK-cell cytotoxicity within the TME [10]. In PDAC, characterized by a highly fibrotic and immunosuppressive stroma, the mechanisms leading to NK cell dysfunction remained poorly defined. Through single-cell transcriptomic profiling, we identified a tumor-associated NK-cell (TaNK) subset exhibiting functional exhaustion. Subsequent investigations revealed that myCAFs served as a key regulator and potentially suppressed NK cell cytoxicity in the TME through the collagen signaling. Specifically, the COL1A2–CD44 axis primarily restricts NK-cell infiltration by enhancing matrix stiffness and hindering immune-cell migration, whereas the COL1A1–LAIR1 axis directly dampened NK cell cytotoxicity through inhibitory collagen binding [37, 39, 40]. Notably, spatial transcriptomic analyses directly validated the spatial distribution characteristics of these molecular interactions. myCAFs and TaNKs consistently colocalized in PDAC tumor regions, and their proximity strongly correlated with reduced NK-cell cytotoxicity. Moreover, the spatial enrichment of COL1A2–CD44 and COL1A1–LAIR1 ligand–receptor colocalization in the same niches further corroborated their synergistic role in shaping an NK-suppressive microenvironment. Complementary multiplex immunofluorescence further confirmed the spatial co-residence of POSTN^+^ myCAFs and CD56^+^ NK cells. Fingdings indicated that NK cells in close contact with myCAFs exhibiting reduced GZMB expression, though direct functional validation was still imperative. Together, these findings revealed a dual mechanism—physical exclusion via COL1A2–CD44 and functional inhibition through COL1A1–LAIR1—that promoted immune evasion in PDAC.

Although exhibiting high tumor mutational burden and abundant neoantigens, PDAC tumors enriched with both myCAFs and TaNK cells showed markedly reduced lymphocyte infiltration, diminished TCR diversity, and elevated immune exclusion scores. This paradox suggested that, despite their intrinsic immunogenicity, these tumors fail to effectively mount anti-tumor immunity. This pattern aligned with the immune-excluded phenotype, where dense stromal components physically and biochemically impede immune-cell migration and activation [41]. Correspondingly, patients in the myCAF_high&TaNK_high group displayed significantly reduced responses to PD-L1 blockade, highlighting the interplay between stromal fibrosis and immune suppression in driving therapeutic resistance. The immunological landscape of solid tumors can be broadly categorized into three distinct phenotypes: immune-inflamed, immune-excluded, and immune-desert types [36]. The immune-inflamed phenotype features abundant effector immune infiltration and active cytokine signaling, typically indicating favorable immunotherapy outcomes. Conversely, the immune-desert phenotype lacks both immune infiltration and antigen presentation, reflecting profound immune ignorance. The immune-excluded phenotype (as exemplified by the myCAF_high&TaNK_high PDAC in this study) is characterized by immune cells accumulating in the peritumoral stroma yet being unable to infiltrate the tumor parenchyma due to structural and signaling barriers. Our spatial and transcriptomic analyses revealed that collagen remodeling by myCAFs, together with TaNK-mediated cytotoxic dysfunction, constituted a critical stromal–immune axis sustaining the immune-excluded state in PDAC.

Despite integrating Mendelian randomization, single-cell and spatial transcriptomics, and multiplex immunofluorescence validation, this study has several limitations. Firstly, it is necessary to validate the inhibitory effect of myCAF-derived collagen on NK-cell activity through in vitro or in vivo experiments. Secondly, the spatial transcriptomic resolution, though informative, may not fully capture fine-grained cell–cell contact or signaling gradients within dense PDAC stroma. Thirdly, the analyzed clinical cohorts were retrospective with limited sample size, necessitating prospective validation in larger patient populations. These limitations highlight the need for further mechanistic and functional studies to validate the proposed stromal–immune interactions.

In summary, this study revealed a stromal–immune regulatory axis in PDAC, where myCAF-derived collagen signaling impaired NK-cell cytotoxicity and contributed to an immune-excluded TME. Through integrated multi-modal transcriptomic analyses, we identified TaNKs exhibiting functional exhaustion and spatial proximity to myCAFs. This mechanism involved both COL1A2–CD44 and COL1A1–LAIR1 signaling pathways. These interactions jointly restrict NK-cell infiltration and suppress their cytotoxic activity, providing a mechanistic basis for immune evasion and immunotherapy resistance in PDAC. These results emphasized that the fibrotic stroma served not merely as a structural component but also as an active regulator of antitumor immunity. Targeting stromal remodeling or disrupting collagen–receptor interactions may restore NK-cell function and enhance immunotherapeutic efficacy. Therapeutic strategies to reinvigorate suppressed NK-cell immunity—by modulating myCAF activity or combining NK-cell activation with immune checkpoint inhibitors—could offer promising avenues for improving the prognosis of this highly refractory malignancy.

## Electronic supplementary material

Below is the link to the electronic supplementary material.

Supplementary material 1

Supplementary material 2

## Data Availability

The datasets employed in this study are available in online repositories. Details regarding repositories and corresponding accession numbers can be found in the article.

## Abbreviations

PDAC: Pancreatic ductal adenocarcinoma
TME: Tumor microenvironment
NK: Natural killer
TaNK: Tumor-associated NK
myCAFs: Myofibroblastic cancer-associated fibroblasts
ECM: Extracellular matrix
scRNA-seq: Single-cell RNA sequencing
GO: Gene ontology
KEGG: Kyoto Encyclopedia of Genes and Genome
OS: Overall survival
