# Supplementary material for: Multimodal transcriptomics reveal myCAF-derived collagen signaling associated with impaired NK-cell cytotoxicity and immune exclusion in pancreatic cancer
Source: J Transl Med. 2026 May 22;24:1061. doi: 10.1186/s12967-026-08237-4 (PMC13483683; doi:10.1186/s12967-026-08237-4)
Supplement: Supplementary file 1 [file 12967_2026_8237_MOESM1_ESM.docx]

**Supplementary Material**

NK cell–derived risk model identifies impaired cytotoxic pathways associated with poor prognosis in pancreatic cancer.

**Supplementary Fig.1** NK cell–derived risk model identifies impaired cytotoxic pathways associated with poor prognosis in pancreatic cancer.

(A) t-SNE visualization of 13,970 single cells from five primary PDAC samples, showing major cell types.
(B) t-SNE plot identifying distinct clusters and representative marker genes (FGFBP2, KLRF1, KLRB1, KLRC1) for the NK cell subset.

(C) LASSO regression analysis.

(D) Forest plot for multivariate Cox regression.

(E) Kaplan–Meier survival curves in the TCGA and GEO cohorts showing that patients in the high-risk group had significantly poorer survival (TCGA, p < 0.001; GEO, p = 0.039).

(F) AUC at 1-, 2-, and 3-years for TCGA and GEO cohorts.

(G) Gene Ontology (GO) enrichment and Kyoto Encyclopedia of Genes and Genomes (KEGG) analysis

(H) Gene Set Enrichment Analysis (GSEA) revealed that the activity of the NK cell–mediated cytotoxicity pathway was markedly reduced in the high-risk group.
